# Contrast-enhanced [^18^ F] fluorodeoxyglucose-positron emission tomography/computed tomography in clinical oncology: tumor-, site-, and question-based comparison with standard positron emission tomography/computed tomography

**DOI:** 10.1186/1470-7330-14-10

**Published:** 2014-04-22

**Authors:** Silvia Morbelli, Raffaella Conzi, Claudio Campus, Giuseppe Cittadini, Irene Bossert, Michela Massollo, Giuseppe Fornarini, Iolanda Calamia, Cecilia Marini, Francesco Fiz, Chiara Ghersi, Lorenzo E Derchi, Gianmario Sambuceti

**Affiliations:** 1 Nuclear Medicine Unit, IRCCS AOU San Martino- IST, Department of Health Sciences, University of Genoa, Largo R Benzi, 10, Genoa 16132, Italy; 2 Department of Radiology, IRCCS AOU San Martino-IST, Genoa, Italy; 3 Italian Institute of Technology (IIT), Genoa, Italy; 4 Department of Medical Oncology, IRCCS AOU San Martino- IST, Genoa, Italy; 5 Institute of Molecular Bioimaging and Physiology, CNR, Genoa-Milan, Italy

**Keywords:** PET/CT, Contrast-enhanced PET/CT, ^18^ [F] fluorodeoxyglucose, Head and neck cancer, Gastrointestinal cancer

## Abstract

**Background:**

The present study aimed to evaluate the added value of contrast-enhanced computed tomography (ceCT) in comparison to standard, non-enhanced CT in the context of a combined positron emission tomography (PET)/CT examination by means of a tumor-, site-, and clinical question-based approach.

**Methods:**

Analysis was performed in 202 patients undergoing PET/CT consisting of a multiphase CT protocol followed by a whole-body PET. The Cochran Q test was performed, followed by a multiple comparisons correction (McNemar test and Bonferroni adjustment), to compare standard and contrast-enhanced PET (cePET/CT). Histopathology or clinical-radiologic follow-up greater than 1 year was used as a reference.

**Results:**

cePET/CT showed significantly different results with respect to standard PET/CT in head and neck and gastrointestinal cancer (*P* = 0.02 and 0.0002, respectively), in the evaluation of lesions located in the abdomen (*P* = 0.009), and in the context of disease restaging (*P* = 0.003). In all these clinical scenarios, adding ceCT resulted in a distinct benefit, by yielding a higher percentage of change in patient management.

**Conclusion:**

These data strongly underline the importance of strictly selecting patients for the combined exam. In particular, patient selection should not be driven solely by mere tumor classification, but should also account for the clinical question and the anatomical location of the neoplastic disease, which can significantly impact patient management.

## Background

Since the early 1990s, functional imaging with positron emission tomography (PET) has been the fastest growing diagnostic modality in oncology [1,2]. In particular, PET with [^18^ F] fluorodeoxyglucose (FDG), which exploits the increased glucose uptake and metabolism by the rapidly proliferating cancer cells, has opened a new field in clinical imaging and is widely used for staging, restaging, therapeutic response monitoring, and prognostic evaluation in patients affected by several types of cancers. However, PET imaging alone is unable to provide precise anatomical localization. Moreover, its utility is often limited by the contextual presence of augmented, non-disease-related glucose uptake in several anatomical districts. These findings can range from the physiologically increased uptake in organs, such as the heart, liver, voluntary muscles, and brain, to paraphysiological scenarios, such as FDG hyperaccumulation in skeletal repair sites, in the active ovarian follicle, and within the active bone marrow [3]. Inflammation is per se a common cause of increased glucose use [3,4].

Over the last decades, hybrid imaging, encompassing combined PET and computed tomography (CT) in a single scanner has become commercially available. Its emergence has had a major impact on diagnostic performance of oncology patients [4], enabling the physician to acquire both metabolic and anatomical imaging data in a single diagnostic session [2].

Modern PET/CT scanners incorporate the latest CT technology, thus technically allowing the execution of multiphase, high-quality CT imaging. Recently, there have been several reports of the possible superiority of contrast-enhanced PET (cePET)/contrast-enhanced CT (ceCT) over standard PET/CT in different clinical settings, including disease staging, restaging, presurgical evaluation, and treatment planning of different tumor types [5-10]. However, in the vast majority of PET/CT scans, the CT component is performed with low current setting and without intravenous contrast, its purpose being to allow an attenuation-weighted reconstruction of the PET sinograms and obtain an anatomical correlation of radiotracer distribution. Indeed, the adoption of a low-dose, contrast-free CT protocol has been guided mostly by practical considerations, so as to reduce radiation burden, reduce patient discomfort, and minimize scanning time, thus increasing the number of exams that a center can perform on daily basis. Also, the lack of large prospective trials and the absence of clinical guidelines have prevented a systematic application of this dual-mode imaging using an evidence-based approach.

It has, however, to be considered that the analyses performed in the vast majority of published studies are mainly focused on a tumor-based approach [5-10] in the absence of a more ‘translational’ evaluation of the feasibility of such a powerful, but practically complex, imaging modality. In fact, the potential advantages of executing the CT part of a PET/CT scan with protocol encompassing contrast administration are related to the greater anatomical details given by ceCT, and its improved characterization of millimetric lesions and the delineation of known lesions with respect to surrounding tissues (i.e. the identification of infiltrative behavior). These latter aspects may be more strictly related to each patient’s specific clinical history (site of disease and clinical question) rather than to the mere classification of tumor type. For these reasons, we hypothesized that a more comprehensive approach would help to identify patients that are more likely to benefit from cePET/CT imaging. This approach should account not only for tumor histopathology, but also for the site of known/suspected lesions and for the clinical question. Thus, the aim of the present study was to evaluate the additional diagnostic value of ceCT in comparison to standard, non-enhanced CT in the context of a combined PET/CT examination by means of a tumor-, site-, and clinical question-based approach.

## Methods

### Patients

Between September 2007 and June 2011, 202 patients were referred to our institution for the execution of multimodal PET/CT, including both non-enhanced and contrast-enhanced diagnostic CT. Clinical indication comprised diagnosis of suspected oncologic disease, diagnosis of a suspected recurrence, and staging, restaging, or post-therapy evaluation of a known oncologic disease. All patients accepted the use of imaging data for research purposes by providing written informed consent that was approved by the local regulatory bodies. Patient characteristics are summarized in Table 1. In these patients, the indications for adding a multiphase ceCT to the standard PET/CT exam protocol were: (1) new patients with no state-of-the-art, whole-body staging examination available; (2) equivocal or insufficient results of previous examinations; (3) precise assessment of tumor extent before local radiation therapy; (4) post-therapy assessment of an inpatient who required restaging with both ceCT and PET/CT.

**Table 1:** Patient characteristics

	**Whole patient group**	**Histopathological confirmation**
	**(*n*** **= 202)**	**(*n*** **= 73)**
Age (years)	61.9 ± 14.9	54.2 ± 17.3
Gender (M/F)	120/82	43/30
*Tumor type*^*a,b*^		
Lung cancer	38	30
Head and neck cancer	22	14
Gastrointestinal cancer^c^	31	7
Breast cancer	21	4
Lymphoma	36	9
Melanoma	19	2
Multiple melanoma	15	5
Other^d^	18	2
*Clinical question*^*b*^		
Diagnosis	22	10
Staging	20	11
Restaging^e^	33	18
Response to chemotherapy	58	6
Response to radiotherapy	18	6
Post-surgery evaluation	11	0
Surveillance	40	22

Values are mean ± standard deviation.

^a^Two patients were not affected by any oncologic disease after histologic confirmation.

^b^Tumor type and clinical questions correspond to the groups submitted to the tumor-based and question-based analyses.

^c^Including esophageal, gastric, and colorectal cancer.

^d^Gynecologic malignancy (*n* = 8), testicular cancer (*n* = 2), sarcoma (*n* = 3), low-differentiated neuroendocrine carcinoma (*n* = 4), thymoma (*n* = 1).

^e^Suspected relapse or patients with potentially resectable metastatic disease.

### Positron emission tomography/computed tomography scanning

Patients were prepared and standard PET/CT exams were performed according to the European guidelines [11]. Briefly, patients fasted overnight prior to the intravenous administration of FDG; this was performed in a quiet room, with the patient lying in a recumbent position and asked not to move. Blood glucose was measured before tracer injection was administered, to ensure blood glucose levels < 160 mg/dl. The dose of FDG varied between 350 and 450 MBq, depending on the patient’s weight, and was injected through a peripheral vein catheter. To minimize artifacts caused by the presence of radioactive urine in the excretory system, patients were asked to drink 500–1000 ml of water 1 h prior to image acquisition and to void just before the scan. No urinary bladder catheterization was used. Whole-body imaging was performed using a combined PET/CT scanner (BioGraph 16 Hi-Rez PET/CT scanner; Siemens AG, Erlangen, Germany). The technical parameters of the 16-detector row, helical CT scanner included a gantry rotation speed of 0.5 s and a table speed of 24 mm per gantry rotation. The PET component of the combined imaging system had an axial view of 16.2 cm (per bed position), with an interslice spacing of 3.75 mm in one bed position. The transaxial field of view and pixel size of the reconstructed PET images were 58.5 cm and 4.57 mm, respectively, with a matrix size of 128 × 128. Data acquisition started 60 ± 10 minutes after intravenous tracer administration. First, unenhanced, low-dose CT was performed at 140 kV and 40 mA for emission-based attenuation correction, immediately followed by a PET scan, which was executed in three-dimensional (3D) mode, with a 3-min acquisition per bed position. The scan was performed starting from the orbital plane on to the mid-thigh, except for those cases where the clinical history demanded a whole-body, head-to-toes scan (e.g. multiple myeloma or melanoma). Attenuation-corrected PET images were reconstructed by means of an ordered-subset expectation maximization, iterative reconstruction algorithm (three iterations, eight subsets). Finally, diagnostic ceCT was performed for the same axial coverage. The entire ceCT data set was automatically fused with the 3D PET images using the integrated software interface provided by the manufacturer (*syngo* Image Fusion; Siemens AG, Erlangen, Germany) to create contrast-enhanced anatomical images superimposed with FDG uptake. We did not experience significant fusion mismatch as the ceCT was performed immediately after the PET emission scan, while the patient was maintaining the same position.

### Contrast-enhanced computed tomography technique

The CT scan was performed immediately after completion of PET acquisition, planned with the same scout view. In most cases, a pre-contrast diagnostic scan was not acquired and the standard acquisition protocol consisted of an arterial phase scan of the upper abdomen, starting 35 s after the start of contrast injection, followed by a portal phase scan, extended from the skull base to the symphysis pubis, starting 70 s after the administration of the intravenous contrast. The scan parameters were as follows. Arterial phase: slice thickness of 5 mm, pitch 0.8, tube rotation speed 0.5 s, 120 kV, reference 175 mA. A dose modulation system was applied to optimize total exposure according to the patient’s body size; an additional set of 1-mm thick slices was reconstructed to obtain high-resolution, multiplanar reformations. Portal phase: slice thickness 5 mm, pitch 0.8, tube rotation speed 0.5 s, 120 kV, reference 175 mA with the same modulation system; 2-mm thick slices at 1.5 mm intervals were reconstructed for multiplanar reformations.

In selected cases, a delayed scan was performed at equilibrium or in the urographic phase, according to the clinical question. In the follow-up of known lesions or in patients with lymphoproliferative disorders and with previous negative imaging examination, only the portal phase scan was obtained, so as to reduce the dose administered to the patient.

Iodinated contrast medium, with a concentration of 350 mg/ml, was injected using a power injector at a flow rate of 3 ml/s and a dose of 80–130 ml, depending on body weight, followed by 40 ml of saline at the same flow rate. Contrast medium administration protocols and measures aiming to prevent contrast-related adverse effects were performed according to the guidelines of the European Society of Urogenital Radiology [12].

Standard, 5-mm thick images were used for rapid evaluation by the radiologist and for reviewing by the referring physician, while thinner slices were used for multiplanar imaging of vessels, bone (ribs and spine), and for high-resolution scanning of lung and liver lesions.

### Image interpretation

Positron emission tomography, ceCT, and fused images were reconstructed for review on a dedicated computer workstation (*syngo* Image Fusion; Siemens AG, Erlangen, Germany).

Initially, PET/low-dose CT and multiphase diagnostic CT images were evaluated independently by two experienced nuclear medicine specialists and by two experienced CT radiologists, respectively. All readers had access to the patient’s clinical history, but were blinded to the other modality results. Subsequently, fused, cePET/CT images were evaluated in consensus, by the combined team of radiologists and nuclear physicians. This latter consensus report was compared with the previous evaluations to determine the additional value of diagnostic CT on PET/CT image interpretation.

In the evaluation of FDG-PET/CT, a lesion was considered positive whenever it showed a non-physiological increase of FDG uptake. In particular, the diagnosis of a PET-positive lesion was also supported by a maximum standardized uptake (SUV_max_) value of at least 2.5 or by an FDG uptake exceeding the surrounding background tissue, the blood pool radioactivity, or the average liver uptake. However, the differentiation between malignant and benign lesion was not based solely on SUV_max_, as the qualitative assessment of increased FDG uptake areas played a major role in the clinical reporting. For instance, if a lesion showed clearly abnormal focal FDG avidity but displayed a SUV_max_ lower than the 2.5 threshold (e.g. due to partial volume effect in small-sized lesions), that lesion was deemed malignant.

On diagnostic ceCT-only images, detection of a pathologic lesion was performed according to published criteria [13-16]. For example, lymph node (LN) assessment in the neck, thorax, and abdomen was based on size criteria (1-cm short-axis diameter threshold). However, the presence of peripheral low attenuation, suggesting a fatty hilum within a LN, was considered a benign sign, regardless of node size. Finally, criteria such as abnormal enhancement, central necrosis, irregular borders, and the presence of infiltrative behavior were used to characterize soft tissue lesions. Consensus interpretation of the PET/ceCT exam was performed according to the following criteria: (1) positive lesions were diagnosed when an abnormal area of focal FDG uptake, as observed in PET images, corresponded to an abnormal finding on CT; (2) LNs with increased glucose uptake were deemed positive for metastatic spread even if they were smaller than 1 cm in short-axis diameter; (3) conversely, LNs with no detectable tracer uptake were deemed negative for metastatic spread, even if they were larger than 1 cm in short-axis diameter; (4) in all the remaining cases, the two readers decided to emphasize either functional information from the PET or morphological information from the ceCT on a case-by-case basis, according to type and site of disease and in relation to the patient’s clinical history. For example, in cePET/CT images, small pathological lesions, such as millimetric lung metastases, which often lack FDG accumulation, were evaluated on the basis of the ceCT results.

### Standard of reference

The final diagnosis was obtained from the results of the histopathologic examination, obtained following surgery or biopsy (73 patients, 147 lesions), or clinical/radiological 12-month follow-up or, again, on the basis of tumor marker levels or on the evolutionary pattern of known findings at subsequent imaging (129 patients, 450 lesions). Follow-up information included physical examination, laboratory tests, tumor markers, other independent imaging studies, such as multislice CT, magnetic resonance imaging, FDG-PET/CT, 18 F-NaF PET/CT, X-ray studies, and bone scans.

The criteria used as the standard of reference were: (1) laboratory findings, such as increasing tumor markers; (2) combination of either negative follow-up imaging findings and negative clinical findings or positive clinical findings with decreasing lesion size after therapy, as determined by subsequent imaging studies; (3) increasing lesion size or metabolic activity in the course of follow-up; (4) subsiding of pathological findings on follow-up PET/CT studies combined with negative clinical follow-up.

### Statistical analysis

Statistical analysis was carried out using the ‘R’ software program [17] and the DiagnosisMed software package [18].

We performed patient-based and lesion-, site-, tumor-, and question-based analyses of the cePET/CT results compared with PET/CT. Tables 1 and 2 list the tumor type, clinical question, and site of disease that were used in the tumor-, question-, and site-based analyses.

**Table 2:** Distribution of lesions

	**Lesions included in the analysis**	**Histopathological confirmation**
	**(*n*** **= 597)**	**(*n*** **= 147)**
*Tumor type*^*a,b*^		
Lung cancer	104	40
Head and neck cancer	61	32
Gastrointestinal cancer^c^	129	24
Breast cancer	60	6
Lymphoma	125	20
Melanoma	26	10
Multiple melanoma	32	3
Other^d^	60	12
*Clinical question*^*b*^		
Diagnosis	22	10
Staging	20	11
Restaging^e^	33	18
Response to chemotherapy	58	6
Response to radiotherapy	18	6
Post-surgery evaluation	11	0
Surveillance	40	22
*Site of disease*^*b*^		
Neck	93	33
Thorax	110	49
Abdomen	220	45
Skeleton-bone marrow	174	20
Lymph nodes^f^	198	32

Values are mean ± standard deviation.

^a^Two patients were not affected by any oncologic disease after histologic confirmation.

^b^Tumor type, clinical questions, and disease sites correspond to the groups submitted to the tumor-based and question-based analyses.

^c^Including esophageal, gastric, and colorectal cancer.

^d^Gynecologic malignancy (*n* = 8) , testicular cancer (*n* = 2), sarcoma (*n* = 3), low-differentiated neuroendocrine carcinoma (*n* = 4), thymoma (*n* = 1).

^e^Suspected relapse or patients with potentially resectable metastatic disease.

^f^Lymph nodes were computed twice: according to their position in the body and independently from their position.

A lesion was included in the analysis if at least one of the three modalities (cePET/CT, ceCT, PET/CT) deemed it positive or if it was considered as non-pathologic at these three modalities but resulted positive at a subsequent histopathologic analysis.

Sensitivity, specificity, positive predictive value, negative predictive value (NPV), accuracy, likelihood ratios, diagnostic odds ratio, error rate, and Youden’s index were calculated using standard statistical formulae, and the 95% confidence interval was determined for each parameter. Differences among imaging modalities were assessed with the Cochran Q test, followed by multiple comparisons using the McNemar test with continuity correction and Bonferroni adjustment. Probability values inferior to 0.05 were considered as statistically significant. The value of adding a diagnostic CT to the standard PET/CT protocol was also evaluated in terms of the impact on patient management, following the information derived from the cePET/CT exam only. This evaluation was performed on a patient basis for each tumor type and clinical question.

In order to better characterize the additional value of ceCT with respect to the low-dose PET/CT study, the management-changing findings were classified into the following categories: (1) metabolism-related; (2) site-related; (3) dimension-related; (4) related to local infiltration or additional findings on ceCT.

Category 1 referred to the improved characterization of a lesion that presented no increment in FDG uptake or whose uptake did not reach the significance threshold. Category 2 encompassed the scenario of a more accurate interpretation of lesions due to improved localization of abnormal FDG uptake, which also allowed a better distinction between abnormal and physiological FDG uptake. Category 3 was applied to the cases of identification of pathologic lesions with size falling below PET image resolution. Finally, category 4 referred to the evaluation of infiltrative behavior or to meaningful findings that had gone undetected at the standard PET/CT scan.

## Results

### Overall diagnostic accuracy and patient-based analysis

Positron emission tomography/CT, ceCT, and combined cePET/CT correctly classified (true positive plus true negative) 173, 153, and 179 patients, respectively. The Cochran Q test evidenced a significant difference in the comparison of the three techniques (Cochran Q = 26.08, degrees of freedom (df) = 2, *P* = 2 × 10^-6^). However, a pairwise comparison using continuity-corrected McNemar tests with Bonferroni adjustment revealed that cePET/CT was not significantly different with respect to PET/CT alone (McNemar chi-squared test = 1.5625, df = 1, *P* = 0.21). Although not reaching significance, cePET/CT presented better sensitivity, NPV, negative likelihood ratio (NLR) and Youden’s index, when compared to PET/CT. Patient-based performance comparisons between PET/CT and combined cePET/CT are shown in Table 3.

**Table 3:** Patient-based performance comparisons between PET/CT and cePET/CT

	**PET/CT**	**cePET/CT**
Sensitivity (%)	92.79 (86.42–96.30)	98.20 (93.67–99.50)
Specificity (%)	76.92 (67.28–84.38)	76.55 (67.02–84.76)
Positive predictive value (%)	83.06 (75.49–88.65)	83.85 (76.56–89.18)
Negative predictive value (%)	89.74 (81.05–94.71)	97.22 (90.43–99.23)
Positive likelihood ratio	4.02 (2.75–5.88)	4.26 (2.92–6.20)
Negative likelihood ratio	0.09 (0.05–0.18)	0.02 (0.01–0.09)
Diagnostic odds ratio	41.67 (16.96–115.67)	174.43 (41.26–1593.39)
Error rate (%)	14.36 (10.19–19.86)	11.39 (7.71–16.51)
Accuracy (%)	85.64 (80.14–89.81)	88.61 (83.49–92.29)
Youden’s index	0.6972 (0.700–0.6940)	0.7512 (0.7541–0.7483)

PET, positron emission tomography; CT, computed tomography; cePET/CT, contrast-enhanced PET/ CT.

### Overall lesion-based analysis

A total of 597 lesions were detected. Among these, 431, 385, and 467 lesions were correctly evaluated (true positive plus true negative) by PET/CT, ceCT and combined cePET/CT, respectively. The Cochran Q test evidenced a significant difference between the three techniques (Cochran Q = 58.3969, df = 2, *P* < 10^-12^). A pairwise comparison using continuity-corrected McNemar tests with Bonferroni adjustment revealed that the evaluation with cePET/CT yielded significantly different results with respect to PET/CT (McNemar chi-squared test = 30.96, df = 1, adjusted *P* < 0.05). Performance comparisons between PET/CT and cePET/CT are shown in Table 4; cePET/CT showed better sensitivity, NPV, NLR, and Youden’s index compared to PET/CT. However, PET/CT showed a slightly better specificity than cePET/CT.

**Table 4:** Lesion-based analysis: performance comparisons between PET/CT and cePET/CT

	**PET/CT**	**cePET/CT**
Sensitivity (%)	80.54 (76.41–84.10)	91.38 (88.25–93.74)
Specificity (%)	54.45 (47.37–61.36)	50.26 (43.24–57.28)
Positive predictive value (%)	78.99 (74.80–82.63)	79.61 (75.72–83.02)
Negative predictive value (%)	56.83 (49.59–63.79)	73.28 (65.12–80.12)
Positive likelihood ratio	1.77 (1.50–2.09)	1.84 (1.58–2.13)
Negative likelihood ratio	0.36 (0.28–0.45)	0.17 (0.12–0.24)
Diagnostic odds ratio	4.93 (3.39–7.21)	10.63 (6.85–16.84)
Error rate (%)	27.81 (24.36–31.53)	21.78 (18.65–25.26)
Accuracy (%)	72.19 (68.47–75.64)	78.22 (74.74–81.35)
Youden’s index	0.349 (0.352–0.347)	0.416 (0.418–0.414)

Estimated parameters corresponding to each technique are presented with a 95% confidence interval shown within parentheses. PET, positron emission tomography; CT, computed tomography; cePET/CT, contrast-enhanced PET/ CT.

### Tumor-based lesion analysis

Table 5 lists the different types of tumor in which lesion-based performance comparison was executed between PET/CT and cePET/CT. The latter imaging modality showed significantly different results with respect to standard PET/CT in head and neck cancer (McNemar chi-squared test = 5.9, df = 1, *P* = 0.02) and gastrointestinal cancer (McNemar chi-squared test = 13.1, df = 1, *P* = 0.0002) patients. Table 6 lists the corresponding sensitivity and specificity values.

**Table 5:** **Statistical difference (*P *** **values) between the performance of PET/CT and cePET/CT for each analyzed tumor type, site of disease, and clinical question**

	***P *** **value**
*Tumor type*	
Lung cancer	0.12
Head and neck cancer	0.02
Gastrointestinal cancer^a^	0.0002
Breast cancer	0.09
Lymphoma	0.24
Melanoma	0.16
Multiple melanoma	0.08
Other^b^	0.33
*Site of disease*	
Neck	0.096
Thorax	0.36
Abdomen	0.009
Skeleton-bone marrow	0.33
Lymph nodes	0.44
*Clinical question*	
Diagnosis	0.39
Staging	0.09
Restaging^c^	0.003
Response to chemotherapy	0.13
Response to radiotherapy	0.1
Post-surgery evaluation	0.54
Surveillance	0.51

^a^Including esophageal, gastric, and colorectal cancer.

^b^Gynecologic malignancy *n* = 8 , testicular cancer *n* = 2, sarcoma *n* = 3, low-differentiated neuroendocrine carcinoma *n* = 4, thymoma *n* = 1.

^c^Suspected relapse or patients with potentially resectable metastatic disease.

PET, positron emission tomography; CT, computed tomography; cePET/CT, contrast-enhanced PET/ CT.

**Table 6:** Performance of PET/CT and cePET/CT in tumors, sites of disease, and clinical questions whose diagnostic results were significantly different between the two imaging modalities

	**PET/CT**	**cePET/CT**
*Tumor type*		
**Head and neck cancer**		
Sensitivity (%)	71.33 (63.64–77.97)	82.67 (75.81–87.89)
Specificity (%)	46.30 (33.69–59.39)	48.15 (35.39–61.15)
**Gastrointestinal cancer**^**a**^		
Sensitivity (%)	71.25 (60.54–80.01)	95.00 (87.84–98.04)
Specificity (%)	84.21 (62.43–94.48)	89.47 (68.61–97.06)
*Site of disease*		
**Abdomen**		
Sensitivity (%)	97.53 (91.44–99.32)	92.59 (84.77–96.56)
Specificity (%)	46.15 (23.21–70.86)	92.31 (66.69–98.63)
*Questions*		
**Restaging**^**b**^		
Sensitivity (%)	83.96 (75.81–89.74)	96.23 (90.70–98.52)
Specificity (%)	78.37 (52.33–92.50)	78.57 (52.41–92.43)

Estimated parameters corresponding to each technique are presented with 95% confidence interval within parentheses.

^a^Including esophageal, gastric, and colorectal cancer.

^b^Suspected relapse or patients with potentially resectable metastatic disease.

PET, positron emission tomography; CT, computed tomography; cePET/CT, contrast-enhanced PET/ CT.

### Site-based analysis of lesions

Five anatomical districts, including neck, thorax, abdomen, skeletal-bone marrow, and LNs (regardless of body region), were evaluated in each patient. Contrast-enhanced PET/CT results were significantly different with respect to standard PET/CT in the abdominal region (McNemar chi-squared test = 6.8, df = 1, *P* = 0.009). As expected, in the subset of patients with abdominal lesions, the largest subgroup was represented by patients with colorectal cancer (*n* = 21) followed by non-Hodgkin lymphoma patients (*n* = 12), while subgroups comprising patients affected by other tumor types were smaller (five esophageal, five breast, two ovarian, and four lung cancers, two melanomas, one neuroendocrine tumor). Table 6 lists the corresponding sensitivity and specificity values.

### Question-based analysis of lesions

Table 1 lists the different clinical questions, submitted independently from the lesion-based performance comparisons between PET/CT and cePET/CT. The results of cePET/CT were significantly different with respect to standard PET/CT when the scenario of disease restaging was considered (McNemar chi-squared test = 8.5, df = 1, *P* = 0.003). Again, among patients submitted for restaging, the largest subgroup of patients was represented by patients with colorectal cancer (*n* = 14) while all other patient subgroups were smaller (three head and neck, three breast, four lung and one ovarian cancers, five melanomas, three non-Hodgkin lymphomas). Table 6 lists the corresponding sensitivity and specificity values.

### Impact on patient management

Findings that were detected only at the cePET/CT imaging modality resulted in a change of management for 15 of the 202 patients (7.4%). In particular, three patients initiated a previously unplanned therapy, six patients avoided inappropriate surgery, while a previously decided medical treatment was spared in four patients. In two patients, the use of cePET/CT significantly modified the radiotherapy protocol. Adding ceCT produced more distinct benefits, i.e. yielded a greater percentage of clinical management modifications in selected tumor types and clinical questions. In fact, the incremental value of this diagnostic technique rose to 22% in patients with head and neck cancer (5/23) and in 16% of patients with gastrointestinal cancer (5/31). Finally, when patients were grouped using the clinical question criterion, the additional value of diagnostic ceCT was more evident in the disease restaging setting (8/33 patients, 24.2%). These patients, submitted to cePET/CT for restaging purposes, were respectively affected by head and neck cancer (*n* = 2), gastrointestinal cancer (*n* = 2), lung cancer (*n* = 1), non-Hodgkin lymphoma (*n* = 1), breast cancer (*n* = 1), and poorly differentiated neuroendocrine carcinoma (*n* = 1).

**Figure 1:**
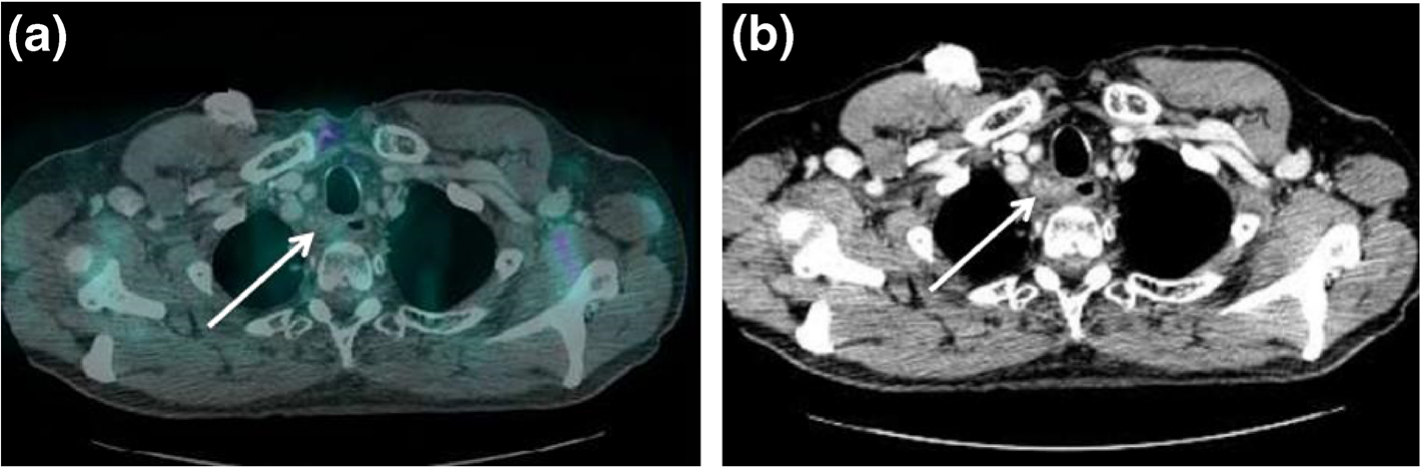
**A 65-year-old male patient affected by pharyngeal cancer with lymph nodal relapse (secondary disease relapse: the first relapse in the neck lymph nodes had been treated with surgery and radiotherapy one year before). (a)** Axial, contrast-enhanced, full-dose CT shows a small lymph node, which seems to infiltrate the upper esophagus. **(b)** As evident from contrast-enhanced PET/CT fused images, this lymph node does not display increased FDG uptake. Subsequent endoesophageal ultrasound biopsy confirmed the presence of lymph node metastasis. The patient was then submitted to chemotherapy. CT, computed tomography; PET, positron emission tomography; FDG, [^18^ F] fluorodeoxyglucose.

**Figure 2:**
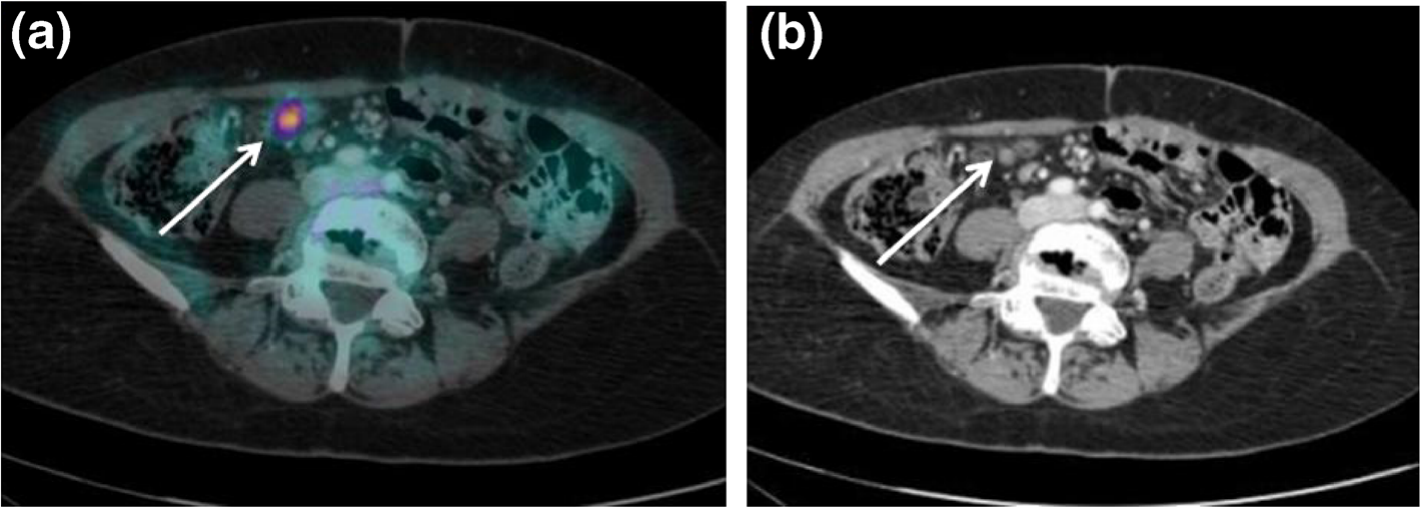
**A 54-year-old female patient previously submitted for surgical treatment for primary colon cancer and with a known, single liver metastasis.** This patient underwent contrast-enhanced PET/CT for the exclusion of other metastatic lesions, as she was a candidate for surgical resection of the hepatic localization. **(a)** Axial, contrast-enhanced, full-dose CT shows a suspicious 8-mm solid lesion close to the small bowel. **(b)** The lesion is highly FDG-avid on contrast-enhanced PET/CT. However, the pathologic nature of this finding is clearly evident only on contrast-enhanced CT, whereas using standard CT, it could have been deemed as unspecific bowel FDG-activity. This patient thus avoided inappropriate surgical treatment and was referred for chemotherapy. PET, positron emission tomography; CT, computed tomography; FDG, [^18^ F] fluorodeoxyglucose.

**Figure 3:**
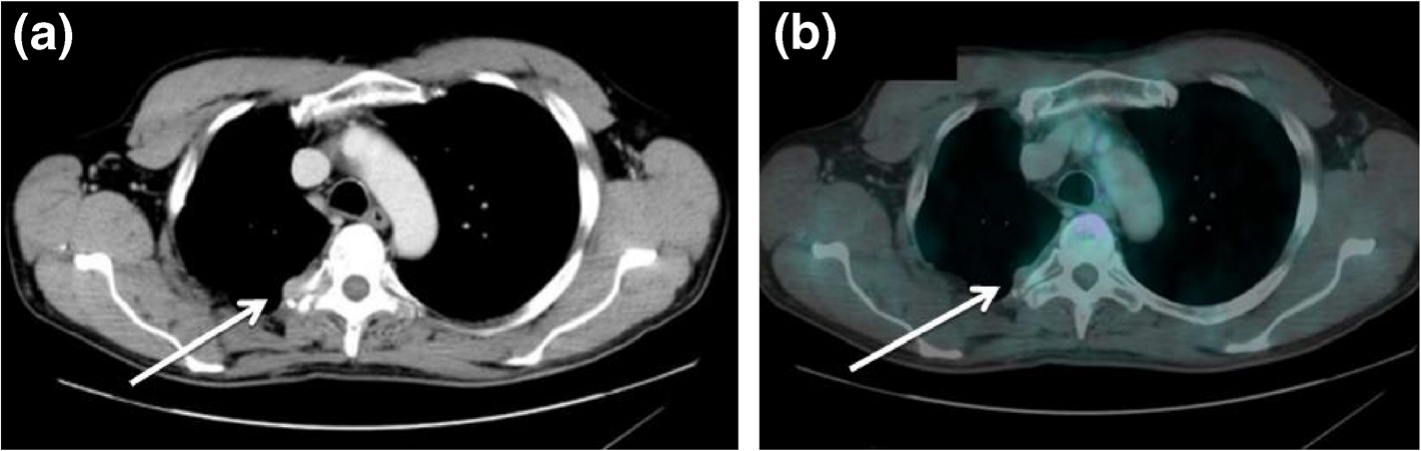
**A 51-year-old male patient, presenting tumor marker increase after surgery for lung cancer. (a)** Axial, contrast-enhanced, full-dose CT showed a paravertebral lesion, which was slightly enlarged and significantly more hyperemic with respect to a previous CT scan. **(b)** The lesion showed very mild FDG uptake; it was, however, classified as highly suspicious on the basis of the contrast-enhanced CT component of the exam. Biopsy confirmed the presence of lung adenocarcinoma metastasis. Accordingly, the patient was referred for radiotherapy. CT, computed tomography; FDG, [^18^ F] fluorodeoxyglucose.

The 15 patients whose management was changed by cePET/CT, were classified according to the previously explained management-changing findings categorization, as follows: category 1 = six patients; category 2 = three patients; category 3 = five patients; category 4 = one patient. Representative cases are shown in Figures 1, 2, and 3.

## Discussion

The present analysis does not support the routine use of cePET/CT. In fact, our study confirms that, when clinically indicated, PET/CT executed with low-dose CT is adequate for the workup of FDG-avid tumor types [2]. However, our analysis demonstrated that ceCT could improve the diagnostic potential of hybrid imaging in specific clinical scenarios, where it can significantly impact patient management.

In particular, patients affected by head and neck or gastrointestinal cancer, and patients undergoing PET/CT for evaluation of abdominal lesions and for restaging purposes, seem to receive a greater benefit from imaging protocols including both PET and ceCT. A greater diagnostic accuracy of cePET/CT has been indeed reported in both head and neck and gastrointestinal cancer patients, with sensitivity and specificity comparable with our results.

### Head and neck cancer

Haerle et al. demonstrated that cePET/CT is superior to PET/CT with regard to pathologically confirmed N0 versus N + status in head and neck cancer patients [19]. In this study, sensitivity and specificity for correct N classification were respectively 70.7% and 50% for PET/CT and 85.5% and 45.5% for cePET/CT. Similarly, Yoshida et al. reported the superiority of cePET/CT over PET/CT in the detection of head and neck malignancies [20]. Overall, these results are consistent with the fact that the interpretation of FDG-PET/CT findings in the neck can be challenging because of the numerous areas of physiologic FDG uptake and also due to the frequently observed pitfalls in post-treatment PET/CT imaging (e.g. slight homogeneous FDG uptake at the tracheostomy site or due to post-treatment edema of the mucosal surfaces). Besides the complexity of neck anatomy, other aspects related to each specific patient can also be advocated. In fact, Haerle et al. highlighted a statistically significant correlation between SUV_max_ and the degree of necrosis in the involved neck LNs [19]. Actually, while the presence of central necrosis in neck LNs is considered as a reliable sign of LN metastasis at ceCT, these nodes are often disregarded by non-enhanced PET/CT because of the lack of FDG uptake in the necrotic, hypocellular tissue. The presence of necrotic LNs can be predicted in some cases, being more frequent in human papillomavirus-associated head and neck squamous cell carcinoma [19]. Accordingly, in the present study, more than one-third of patients whose management was changed by cePET/CT had PET-negative or only faintly positive lesions, whose presence could have been predicted before the exam. This finding confirms that patients can be selected for cePET/CT on the basis of their specific clinical history.

### Gastrointestinal cancer and abdominal imaging

Dirisamer et al. showed that the sensitivity and specificity of PET/CT in colorectal cancer rose from 85 to 100% and from 70 to 81%, respectively, when contrast medium was added to the CT component of the exam [21]. More importantly, these authors showed that the significant increase in sensitivity (which is similar to the one highlighted in the present study) was due to a misdetection of 67% of the metastases at the standard PET/CT exam. Interestingly enough, these metastases were smaller than 8 mm in the majority of cases (94%). Indeed, small metastatic lesions from colon cancer, located within the liver or in the lung parenchyma, can show absent or only modest tracer uptake at PET/CT. Contrast enhancement is particularly effective when evaluating lesions within the liver, as it allows the spotting and classification of lesions as positive, small, and faintly FDG-avid, otherwise missed by standard PET/CT. In this type of patient, other reasons for false-negative PET/CT results are millimetric peritoneal metastatic spread or small peritoneal lesions located in proximity to areas of non-specific FDG uptake, which are common in the intestinal tract [22]. In the present study, the potential usefulness of cePET/CT in the evaluation of patients with gastrointestinal cancer is further testified by the fact that patients with colorectal cancer represented the largest subgroup both in patients with abdominal lesions and in patients submitted for restaging. In these two groups, we independently demonstrated a significantly greater diagnostic accuracy of cePET/CT with respect to standard PET/CT.

### Restaging in suspected cases or known disease relapse

Both these findings (tiny lesions or lesions close to sites of physiologic FDG uptake), whenever undetected by PET/CT, may significantly undermine the correct management of patients. Accordingly, these reasons account for more than one-half of cePET/CT findings that demanded a change of therapeutic strategy in patients included in the present study and are indeed especially relevant as they can avoid inappropriate surgery. On the other hand, ceCT alone showed limited capability in differentiating disease recurrence from post-radiotherapy tissue reaction, while PET/CT displayed a good performance in telling apart these two settings, owing to its intrinsic capacity to identify viable tumor tissue [23]. Overall, both very small lesions, which are likely to produce false-negatives at PET/CT, and local, post-therapy changes, which are likely to prove a diagnostic challenge at ceCT alone, are particularly relevant in patients submitted to medical imaging for restaging or for the preoperative workup of metastatic lesions, candidates for surgical resection. Altogether, these findings strongly suggest that restaging of patients with gastrointestinal cancer could be one of the main indications for cePET/CT use.

### Patient selection based on criteria other than tumor type

Despite the advantages of cePET/CT highlighted here and in previous studies, the use of cePET/CT is still not justified for clinical routine examinations due to higher costs, increased radiation burden, and potential adverse drug reaction to the intravenous contrast medium. Moreover, to obtain an accurate fusion of the two image sets, ceCT should be performed without repositioning the patient. This demands the presence of a physician for immediate image assessment after the standard PET/CT examination, thus resulting in a lowered patient throughput, because of additional time requirements for image review [24]. Therefore, given the demands for FDG-PET/CT and the number of available PET/CT scanners, the diffusion of cePET/CT as a ‘one-stop-shop’ examination for all patients submitted to PET/CT does not presently appear to be the most appropriate choice.

In this scenario, our approach pinpoints the situations where cePET/CT could be of benefit, not only on the basis of tumor type, but also according to the criteria related to the site of lesions and to the clinical question. This multiple parameter statistical analysis further defines the subsets of patients that can be candidates for cePET/CT, on the basis of their specific clinical history. The present study suggests that these patients have to be specifically identified, for example, in the context of oncologic, multidisciplinary, disease-management team discussions.

### Limitations

The present study has several limitations. It was a single-center, retrospective study whose results may have been influenced by its study population, including cancer patients with both initial and recurrent disease. Moreover, due to ethical reasons, the histologic verification of metastasis was not performed for all distant lesions. However, as in other studies, the presence of metastasis was verified with a 1-year imaging follow-up, to ensure the highest possible confidence. We should also underline that we did not directly compare the diagnostic performance of PET/CT with separate CT as we focused on the specific added value of ceCT with respect to standard PET/CT. Finally, as many workstations and several freeware software programs allow to fuse PET and ceCT images, and as ceCT is becoming more and more a ‘frontline’ investigation, a further question concerns the different diagnostic accuracy of combined cePET/CT obtained in a single session with respect to the off-line image fusion of PET images with a recently obtained CT scan. This issue, which is crucial and the object of active work and discussion within radiological and nuclear medicine societies, is presently beyond the aims of our study and deserves further specific investigation.

## Conclusion

We have highlighted a significant benefit of adding ceCT to PET/CT hybrid imaging in patients with head and neck or gastrointestinal cancer. This imaging modality was particularly helpful in the setting of disease restaging, in the presence of increased tumor markers, or in the case of metastatic lesions candidate for surgical treatment. Similarly, regardless of tumor type, lesions located within the abdomen were also more correctly classified thanks to the help of diagnostic ceCT. The present study could not highlight such a significant benefit in the general population of oncology patients submitted to standard FDG-PET/CT, which should be preferred when clinically indicated. Accordingly, these data strongly underline the importance of strictly selecting patients for the combined exam. In the age of personalized medicine and multidisciplinary approaches in oncology patients, the present results allow us to propose a clinical, history-related criterion when selecting candidates for cePET/CT, thus allowing the right imaging workup for the right patient without lowering patient throughput and without causing an unjustified increase in radiation burden.

## Competing interests

The authors declare that they have no competing interest.

## Authors’ contributions

SM, RF, GS, GC, LE: data acquisition, study concept and design, analysis and interpretation of the data and text writing. CC: statistics, and interpretation of the data and text writing. CG: FDG production, interpretation of the data and critical revision of the text. GF: study concept and design, interpretation of the data and critical revision of the text. IB, MM, FF, CM and IC: analysis and interpretation of the data and critical revision of the text. All authors read and approved the final manuscript.
